# Type of diet modulates the metabolic response to sleep deprivation in rats

**DOI:** 10.1186/1743-7075-8-86

**Published:** 2011-12-12

**Authors:** Paulo JF Martins, Leandro Fernandes, Allan C de Oliveira, Sergio Tufik, Vânia D'Almeida

**Affiliations:** 1 Department of Psychobiology, Universidade Federal de São Paulo-UNIFESP/São Paulo - Brazil

**Keywords:** Body weight, liquid diet, metabolism, hyperphagia, liver glycogen, hormones, rats, sleep deprivation, energy intake

## Abstract

**Background:**

Evidence suggests that sleep loss is associated with an increased risk of obesity and diabetes; however, animal models have failed to produce weight gain under sleep deprivation (SD). Previous studies have suggested that this discrepancy could be due to more extreme SD conditions in experimental animals, their higher resting metabolic rate than that of humans, and the decreased opportunity for animals to ingest high-calorie foods. Thus, our objective was to determine whether diets with different textures/compositions could modify feeding behavior and affect the metabolic repercussions in SD in rats.

**Methods:**

Three groups of male rats were used: one was designated as control, one was sleep deprived for 96 h by the platform technique (SD-96h) and one was SD-96h followed by a 24-h recovery (rebound). In the first experiment, the animals were fed chow pellets (CPs); in the second, they received high-fat diet and in the third, they were fed a liquid diet (LD).

**Results:**

We observed that SD induces energy deficits that were related to changes in feeding behavior and affected by the type of diet consumed. Regardless of the diet consumed, SD consistently increased animals' glucagon levels and decreased their leptin and triacylglycerol levels and liver glycogen stores. However, such changes were mostly avoided in the rats on the liquid diet. SD induces a wide range of metabolic and hormonal changes that are strongly linked to the severity of weight loss.

**Conclusions:**

The LD, but not the CP or high-fat diets, favored energy intake, consequently lessening the energy deficit induced by SD.

## Introduction

Epidemiological studies have identified a relationship between a reduced number of sleep hours per day and increased body mass index, increased appetite, reduced leptin and augmented ghrelin levels [1-3], corroborating the view that inadequate sleep time is a risk factor for human obesity [2,4,5]. Paradoxically, sleep-deprived rats present a syndrome of increased feeding accompanied by weight loss [6]. Therefore, many studies have sought to identify the complex relationship between energy homeostasis and sleep to solve this paradox.

Rechtschaffen's group characterized a long-term sleep deprivation (SD) response and found a progressive increase in both food intake and energy expenditure [7,8]. During SD, food intake of rats can be 80 to 220% higher than the pre-sleep-loss baseline [8-11]. Consistent with a higher drive for feeding, the hypothalamic expression of the anorexigenic peptide pro-opiomelanocortin is decreased, while levels of the orexigenics prepro-orexin and neuropeptide Y increase after SD [12,13].

SD also induces increased levels of catabolic hormones, such as glucocorticoids, epinephrine and norepinephrine [10,13-15], and reduced levels of anabolic hormones, including leptin, insulin and growth hormone [9,10,16]. Therefore, it is assumed that SD is a stressful condition [17] characterized by weight loss [8-11,16] despite increased food intake [18].

We previously reported that sleep-deprived rats may not consume as much food as they seek [19]. This observation was associated with a remarkable increase in food spillage, mainly resulting from stereotyped gnawing behavior [20]. Furthermore, it is well known that under stress conditions, humans and animals may change their feeding preference to comfort foods, especially foods with high caloric density, to help cope with stressors [21]. Although it has been hypothesized that sleep-deprived rats prefer foods with higher reward values [22], there have been no studies verifying whether diets with different palatability and compositions affect the metabolic consequences of SD.

Previously, we showed that rats fed a liquid diet (LD) increased their food intake after 72 h of SD, which minimized weight loss and other metabolic abnormalities [13]. Moreover, diets rich in fat are known for their rewarding properties. More recently, a study reported that a high-fat cafeteria diet induced dopamine D2 receptor downregulation, which supports the compulsive eating behavior observed in obese rats [23].

Hence, the present study was conducted to determine whether different diets could modify SD-related feeding behavior and its metabolic repercussions in rats. We hypothesized that providing a LD, which does not stimulate gnawing behavior, or providing high-fat diet, which have increased caloric density, could attenuate the metabolic repercussions and improve the knowledge about the paradoxical association between SD and weight loss in the animal model.

## Methods

### Animals, Housing Conditions and Ethical Care

Male Wistar rats from a colony maintained by the Psychobiology Department - UNIFESP were employed. These animals were derived from the Charles River Laboratories, Inc. (Wilmington, MA, USA) foundation colony. Throughout the experiment, all animals were kept on a 12:12 h light-dark cycle (lights on at 0700 h) under controlled temperature conditions (21° - 24°C) and given free access to food and water. Animal care and use procedures were conducted by trained personnel (FELASA Category C) in accordance with the Guide for the Care and Use of Laboratory Animals. The experimental protocol was approved by the Ethical Committee of UNIFESP (CEP n° 0064/01).

### Sleep Deprivation Procedure

The SD procedure was an adaptation of the classical model for use with rodents. This method consists of placing an animal on top of a narrow platform (6.5 cm in diameter) surrounded by water in a 23 × 23 × 35 cm container [for details, see Martins et al. [19]. All animals were allowed to adapt to the platform for 30 to 40 min for three consecutive days, and SD was initiated at 0800 h after a day of washout. Members of the control group were individually placed in the same container as the sleep-deprived animals, but water was substituted for wood shavings. This substitution was also performed during the recovery period in the rebound group. Previous data from our laboratory indicate that this methods results in a complete elimination of paradoxical sleep and a 37% decrease in slow-wave sleep. Moreover, sleep recovery after the 96 h SD protocol is characterized by a increased paradoxical sleep time (+184.7%) accompanied by a reduction in slow-wave sleep (-12.2%) [24].

### Experimental Procedures for Rats Fed with Chow Pellets

**Figure 1:**
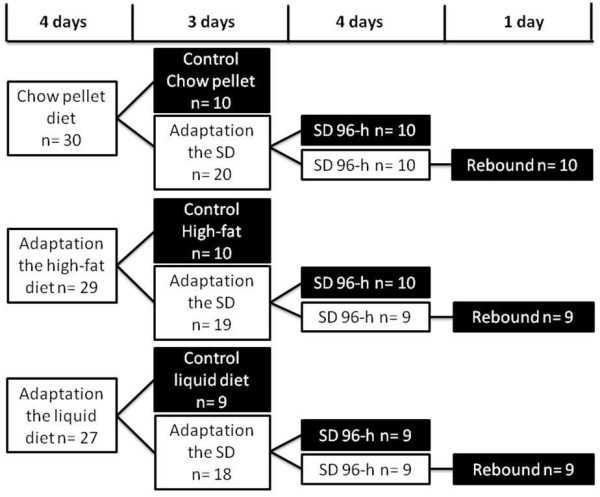
**Overall study design**. SD = sleep deprivation. The black boxes represent the formation of the experimental groups euthanized after the procedure.

Thirty rats were housed for 3 days in individual cages (floor area 0,529 m^2^) and adapted to SD procedures for 3 days, followed by a washout day. Thereafter, the animals were weighed (362.5 ± 39.8 g), and weight-matched cohorts were distributed into the control, sleep-deprived for 96 h (SD-96h), and sleep-deprived for 96 h recovered for 24 h groups (rebound) (see Figure 1). Sleep-deprived and rebound groups were submitted to 96 h of SD, while control rats were kept in isolated home-cages. After 96 h of SD, the rebound group was permitted to sleep for 24 h before sample collection. All animals were euthanized by decapitation between 0700 h and 0930 h for blood and liver sample collection. Stomachs were dissected at the level of the lower gastroesophageal sphincter, and the pylorus and wet stomach weights were registered as an indication of recent food intake. Chow pellets (CPs; 25.3% calories from protein, 11.6% from fat and 63.1% from carbohydrate; 3.485 Kcal/g; Nuvilab-CR1, Colombo, Brazil) were accessible from wall feeders during all experiments. Body weights and the weight of the food removed from the feeders were recorded daily between 0700 and 0800 h. In addition, during SD, the 24-h food intake was calculated, as described elsewhere [19,25].

### Experimental Procedures for Rats Fed a High-Fat Diet

Seven days before the experiment, rats were housed individually and adapted to consume a high-fat diet. Only the animals that recovered their previous body weight and had a stable food intake were used. A group of 29 rats (351.9 ± 22.6 g) were weight matched and distributed among the control, SD-96h and rebound groups (Figure 1). High-fat diets were accessible throughout all experiments in wall feeders, and all other procedures were the same as for the standard experiment. The high-fat diet was prepared by adding peanuts, milk chocolate and sweet biscuits to the standard recipe (17.4% calories from protein, 46.9% from fat, and 35.7% from carbohydrate; 5.0 Kcal/g; Nuvilab-CR1, Colombo, Brazil). In this experiment, we were unable to recover the crumbs from the water to determine food spillage during SD.

### Experimental Procedures for Rats Fed the LD

Twenty seven rats (379.9 ± 37.6 g) were adapted, matched and assigned to groups, as described above (Figure 1). The LD (20.8% protein, 11.9% fat, and 67.3% carbohydrate; 1.0 Kcal/mL; cat. #F1268; Bio-Serv, French-town, NJ, USA) was delivered via feeding tubes (cat. #9011; Bio-Serv) and accessible from wall feeders during all experiment. Food intake and body weight were recorded as described for the CP experiment.

### Analytical Procedures

Blood was collected in tubes containing pre-chilled disodium fluoride, ethylenediaminetetraacetic acid (EDTA), and either heparin or no anticoagulant (Becton Dickinson, New England, UK). The tubes were centrifuged at 4°C for 10 min at 3,000 rpm to extract plasma and serum aliquots. A set of serum aliquots was stored at -80°C for insulin, glucagon, leptin, adiponectin, ghrelin (Linco Research, St Louis, MO, USA) and corticosterone measurements (ICN-Biomedical, Orangeburg, NY, USA) using Radioimmunoassay (RIA) kits. Immediately after collection, plasma and another set of serum aliquots were used for glucose and lactate assays, and total cholesterol, high-density lipoprotein cholesterol (HDL), and triacylglycerol were measured using colorimetric automated procedures (ADVIA 1650, BAYER Diagnostics Corporation) routinely performed in clinical laboratories. Liver samples were rapidly excised, weighed and kept in a potassium hydroxide (30%) solution until glycogen storage was measured by sulfuric acid-antrone reaction on the day of sacrifice, as described elsewhere [26,27].

### Statistical Analysis

The results are presented as means ± SD unless specifically noted as means ± SE. To satisfy the basic assumptions of the analysis of variance (ANOVA), we calculated the percent change of food intake and body weight by normalizing the data to the values recorded 24 h before the baseline, thereby preserving the variance within groups for all timepoints. Because there was no protocol difference between the SD and rebound groups except for the rebound group's day of recovery after 96 h of sleep deprivation, we considered both groups together as a single sleep-deprived group and compared the body weight and food intake of this group with those of the control group using a two-way ANOVA. To verify the effects of rebound on body weight and food intake, a one-way ANOVA was performed to independently compare the rebound group before and after sleep deprivation and after 24 h of recovery. For metabolites and hormonal parameters, we used a one-way ANOVA for each experiment. All ANOVAs were followed by Tukey post-hoc tests, with the alpha value set at 0.05. The data were analyzed with the STATISTICA 6.0 software.

## Results

### CP diet experiment

**Figure 2:**
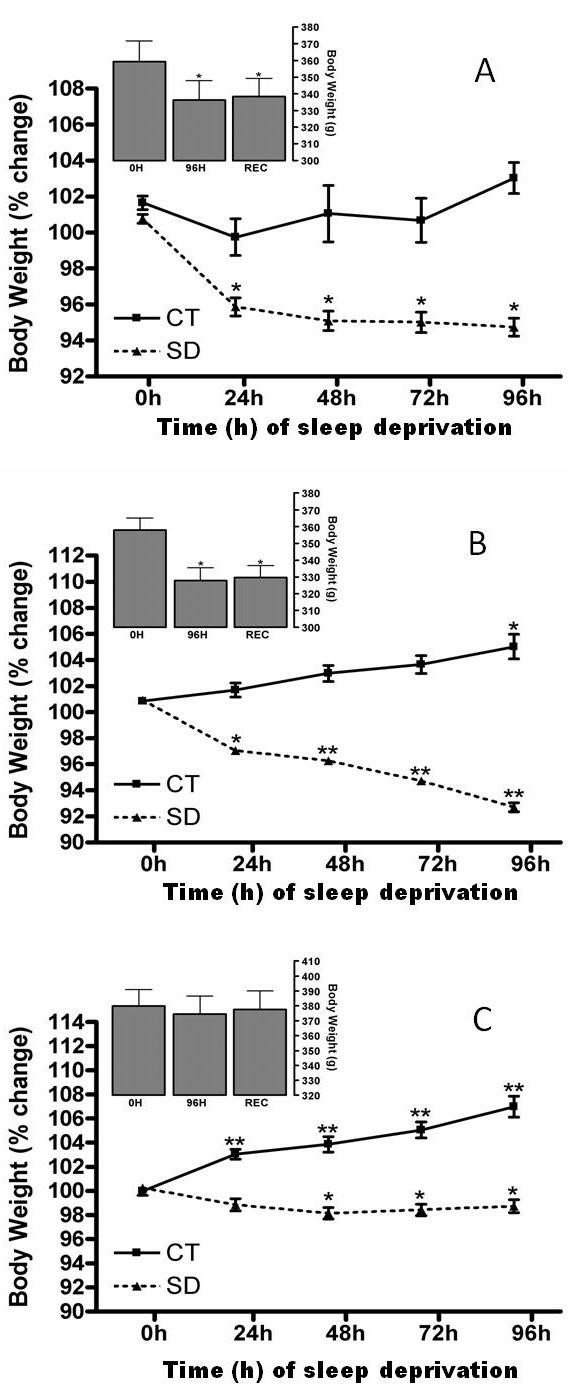
**A) Change in body weight of rats fed a chow pellet diet**. REC = Sleep Recovery. The mean percentage change (mean ± SE) of body weight for control (■; n = 10) and sleep-deprived (SD) (▲; n = 20) rats compared with pre-sleep deprivation (0 h) and 24 h, 48 h, 72 h and 96 h of sleep deprivation. The upper panel shows the mean ± SE body weight (g) at 96 h of sleep deprivation and after 24 h sleep rebound (n = 10). *Different from 0 h, Tukey test, p < 0.05. B) Change in body weight of rats fed with high-fat pellets. REC = Sleep Recovery. The mean percentage change (mean ± SE) of body weight for control (■; n = 10) and sleep-deprived (SD) (▲; n = 19) rats compared to pre-sleep deprivation (0 h) and 24 h, 48 h, 72 h and 96 h of sleep deprivation. The upper panel shows mean ± SE body weight (g) at 96 h of sleep deprivation and after 24 h sleep rebound (n = 9). *Different from 0 h, Tukey test, p < 0.05; **Different from 0 h and SD group, Tukey test, p < 0.05. C) Change in body weight of rats fed a liquid diet. REC = Sleep Recovery. The mean percentage change (mean ± SE) in body weight for control (■; n = 9) and sleep-deprived (SD) (▲; n = 18) rats compared to pre-sleep deprivation (0 h) and 24 h, 48 h, 72 h and 96 h of sleep deprivation. The upper panel shows the mean ± SE body weight (g) at 96 h of sleep deprivation and after 24 h sleep rebound (n = 9). *Different from 0 h, Tukey test, p < 0.05. **Different from 0 h and CT group, Tukey test, p < 0.05.

Body weight decreased after 24 h of SD, compared with the pre-SD day (0 h) and the control rats (CT). This decrease was maintained for 96 h [two-way repeated-measures ANOVA group effect, control *vs*. SD: *F*(1,28) = 24.63, p < 0.0001; group × time interaction effect: *F*(4,112) = 17.16, p < 0.0001] (Figure 2A). After the 24-h rebound period, the decreased body weight was maintained [one-way ANOVA time effect: *F*(2,18) = 66.43, p < 0.0001] (Figure 2A, left upper panel). As observed previously, the largest body weight loss occurred during the first 24 h of SD.

**Figure 3:**
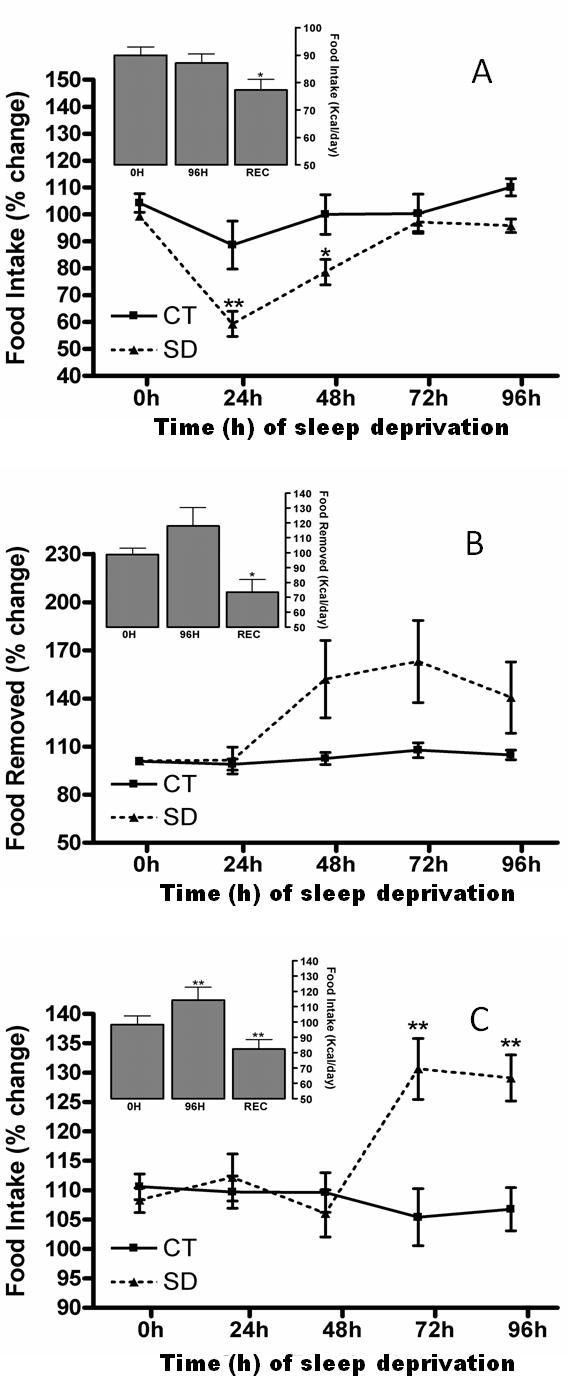
**A) Change in food intake of rats fed a chow pellet diet**. REC = Sleep Recovery. The mean percentage change (mean ± SE) of food intake for control (■; n = 10) and sleep-deprived (SD) (▲; n = 20) rats compared to pre-sleep deprivation (0 h) and 24 h, 48 h, 72 h and 96 h of sleep deprivation. The upper panel shows the mean ± SE food intake (Kcal/day) at 96 h of sleep deprivation and after 24 h sleep rebound (n = 10). *Different from 0 h, Tukey test, p < 0.05; **Different from respective 0 h and time-corresponding SD group, Tukey test, p < 0.05. B) Removed food (%) of rats fed with high-fat pellets. REC = Sleep Recovery. The mean percentage change (mean ± SE) of food removed for control (■; n = 10) and sleep-deprived (SD) (▲; n = 19) rats compared to pre-sleep deprivation (0 h) and 24 h, 48 h, 72 h and 96 h of sleep deprivation. The upper panel shows the mean ± SE food intake (Kcal/day) at 96 h of sleep deprivation and after 24 h sleep rebound (n = 9). **Different from 0 h and SD group, Tukey test, p < 0.05. C) Change in food intake of rats fed a liquid diet. REC = Sleep Recovery. The mean percentage change (mean ± SE) of food intake for control (■; n = 9) and sleep-deprived (SD) (▲; n = 18) rats compared to pre-sleep deprivation (0 h) and 24 h, 48 h, 72 h and 96 h of sleep deprivation. The upper panel shows the mean ± SE food intake (Kcal/day) at 96 h of sleep deprivation and after 24 h sleep rebound (n = 9). *Different from 0 h, Tukey test, p < 0.05.

Food intake also significantly changed throughout the study [two-way repeated-measures ANOVA group effect, control *vs*. SD: *F*(1,28) = 6.63, p = 0.01; group × time interaction effect: *F*(4,112) = 4.31, p < 0.01]. SD rats, in particular, had decreased food intake at 24 h and 48 h compared to 0 h, although after 48 h, their food intake was similar to that of control rats (Figure 3A). Interestingly, during the rebound period [one-way ANOVA time effect: *F*(2,18) = 6.19, p < 0.01], food intake was reduced relative to both 0 h and after 96 h (Figure 3A, left upper panel).

After 96 h of SD, the rats (SD-96h) showed no differences in plasma glucose or lactate compared to control animals [*F*(2,27) = 2.11, p = 0.13; *F*(2,27) = 1.36, p = 0.27, respectively]; however, we observed a fasting-like metabolic profile, in which stomach weight [*F*(2,27) = 8.99, p = 0.001] and liver glycogen storage [*F*(2,27) = 22.39, p < 0.0001] were reduced but plasma ketone body concentrations [*F*(2,27) = 5.50, p < 0.01] were higher than in controls (Table 1). After 24 h of recovery, rebound rats had lower stomach weights and higher ketone body levels than controls, and liver glycogen storage was restored to control levels (Table 1). Rats undergoing SD had reduced triacylglycerol levels [*F*(2,27) = 9.87, p < 0.001] and increased HDL cholesterol [*F*(2,27) = 5.85, p = 0.01]. Triacylglycerol and HDL cholesterol returned to control levels after 24 h of rebound, but total cholesterol remained lower than that of the SD-96h group [*F*(2,27) = 5.98, p < 0.01] (Table 1).

**Table 1:** Metabolic parameters of control, sleep-deprived for 96 h (SD-96h) and recovery (Rebound) rats fed with regular chow pellets

PARAMETERS	CONTROL	SD-96H	REBOUND
Stomach weight (g/100 g of body weight)	2.5 ± 0.84	1.52 ± 0.48*	1.40 ± 0.53*
Liver glycogen (mg/100 mg tissue)	5.27 ± 1.34	1.90 ± 0.87*	4.34 ± 1.23
Ketones bodies (μM)	154.46 ± 34.00	204.05 ± 30.69*	195.15 ± 41.36*
Glucose (mg/dL)	103.70 ± 9.99	107.25 ± 18.32	95.40 ± 9.39
Lactato (mmol/mL)	1.57 ± 0.73	1.81 ± 0.76	1.34 ± 0.29
Total cholesterol (mg/dL)	57.96 ± 6.62	63.13 ± 10.63	49.03 ± 9.52^#^
HDL cholesterol (mg/dL)	28.49 ± 1.78	33.09 ± 5.46*	25.93 ± 5.20
Triacylclycerol (mg/dL)	56.93 ± 17.19	27.45 ± 9.16*	45.06 ± 15.36

Data are means ± SD of 10 rats per group. *Different from control group, Tukey test, p < 0.05; ^#^Different from SD-96h group, Tukey test, p < 0.05.

A SD-induced negative energy balance was also evident from reduced insulin [*F*(2,27) = 3.70, p = 0.03] and leptin [*F*(2,25) = 9.43, p < 0.001] levels, while glucagon [*F*(2,27) = 12.27, p < 0.001], ghrelin [*F*(2,27) = 15.83, p < 0.0001] and corticosterone levels [*F*(2,27) = 14.81, p < 0.0001] were increased (Table 2) in SD-96h rats compared to controls. After 24 h of recovery, rebound rats had restored levels of corticosterone, glucagon and ghrelin, but serum leptin concentrations remained low. There were no differences in adiponectin levels among the three groups [*F*(2,27) = 0.15, p = 0.85] (Table 2).

**Table 2:** Hormonal concentrations of control, sleep-deprived for 96 h (SD-96h) and recovery (Rebound) rats fed with regular chow pellets

PARAMETERS	CONTROL	SD-96H	REBOUND
Insulin (ng/mL)	1.96 ± 1.23	0.91 ± 0.52*	1.68 ± 0.78
Glucagon (ng/mL)	80.54 ± 26.70	122.19 ± 23.00*	76.52 ± 17.91
Ghrelin (μg/mL)	2.40 ± 0.64	3.40 ± 0.91*	1.67 ± 0.41
Leptin (ng/mL)	3.34 ± 1.49	1.54 ± 0.64*	1.63 ± 0.64*
Adiponectin (μg/mL)	2.75 ± 0.76	2.92 ± 1.03	2.93 ± 0.71
Corticosterone (ng/mL)	81.10 ± 41.43	188.60 ± 74.67*	66.90 ± 40.82

Data are means ± SD of 10 rats per group. *Different from control group, Tukey test, p < 0.05.

### High-fat diet experiment

Body weight was decreased after 24 h of SD compared to pre-SD (0 h) and control rats, from 24 h to 96 h. Two-way repeated-measures ANOVA group effect, control *vs*. SD: *F*(1,27) = 86.02, p < 0.0001; group × time interaction effect: *F*(4,108) = 50.84, p < 0.0001] (Figure 2B). After 24 h of rebound, body weight remained reduced [one-way ANOVA time effect: *F*(2,16) = 23.57, p < 0.0001], as observed after 96 h of SD (Figure 2B, left upper panel). As observed previously, the largest body weight loss occurred during the first 24 h of SD.

The amount of food removed from the feeders did not differ throughout the experiment [two-way repeated-measures ANOVA group effect, control *vs*. SD: *F*(1,27) = 2.39, p = 0.13; group × time interaction effect: *F*(4,108) = 1.51, p < 0.20] (Figure 3B). However, during the rebound period, [one-way ANOVA for time effect: *F*(2,16) = 6.70, p < 0.01] food removed was reduced relative to intake at 96 h (Figure 3B, left upper panel).

As with the sleep-deprived rats fed regular CPs, the SD-96h rats fed a high-fat diet similar metabolic effects to those of control animals; however, plasma glucose levels were decreased in the rebound group [*F*(2,26) = 7.32, p < 0.01]. There was a negative energy profile in which stomach weight [*F*(2,26) = 13.83, p < 0.0001] and liver glycogen storage [*F*(2,26) = 18.24, p < 0.0001] were reduced and ketone body concentrations [*F*(2,26) = 6.19, p < 0.01] were increased in SD-96h rats compared to the control group (Table 3). After 24 h of recovery, rebound rats had lower stomach weights, but their ketone levels and liver glycogen were similar to those of the control group (Table 3). Serum lipid analyses revealed no effect of SD on total cholesterol levels [*F*(2,26) = 1.44, p = 0.25]. The SD-96h group had increased HDL cholesterol [*F*(2,26) = 10.73, p < 0.001] and reduced triacylglycerol levels [*F*(2,26) = 10.77, p < 0.001]; these returned to control levels after 24 h of rebound (Table 3).

**Table 3:** Metabolic parameters of control, sleep-deprived for 96 h (SD-96h) and recovery (Rebound) rats fed a high-fat diet

PARAMETERS	CONTROL	SD-96H	REBOUND
Stomach weight (g/100 g of body weight)	1.93 ± 0.48	1.11 ± 0.52*	0.97 ± 0.21*
Liver glycogen (mg/100 mg tissue)	5.47 ± 1.71	1.90 ± 1.15*	4.36 ± 1.07
Ketones bodies (μM)	201.42 ± 93.89	469.91 ± 306.6*	206.75 ± 77.12
Glucose (mg/dL)	105.60 ± 10.46	115.30 ± 11.42	97.33 ± 8.44^#^
Total cholesterol (mg/dL)	77.40 ± 7.04	88.10 ± 14.08	78.44 ± 7.58
HDL cholesterol (mg/dL)	41.10 ± 5.42	52.40 ± 6.53*	44.22 ± 4.63
Triacylclycerol (mg/dL)	237.50 ± 104.31	74.60 ± 33.66*	149.00 ± 80.82

Data are means ± SD of 9-10 rats per group. *Different from control group, Tukey test, p < 0.05; ^#^Different from SD-96 h group, Tukey test, p < 0.05.

The hormonal profile of SD-96h rats fed the high-fat diet was also consistent with a negative energy balance and was characterized by decreased leptin [*F*(2,26) = 8.80, p < 0.01] and increased glucagon [*F*(2,26) = 4,78, p < 0.05], ghrelin [*F*(2,26) = 3.89, p < 0.05] and corticosterone levels [*F*(2,26) = 20.07, p < 0.0001] (Table 4). Following recovery, rebound rats had corticosterone, glucagon and ghrelin levels similar to those of controls, but their serum leptin concentrations remained lower. Insulin [*F*(2,26) = 3.80; p < 0.05] levels increased and adiponectin [*F*(2,26) = 4,86; p < 0.05] decreased in the rebound group (Table 4).

**Table 4:** Hormonal concentrations of control, sleep-deprived for 96 h (SD-96h) and recovery (Rebound) rats fed a high-fat diet

PARAMETERS	CONTROL	SD-96H	REBOUND
Insulin (ng/mL)	0.64 ± 0.31	0.84 ± 0.51	1.09 ± 0.58*
Glucagon (ng/mL)	71.24 ± 16.56	102.19 ± 30.29*	81.08 ± 18.71
Ghrelin (μg/mL)	0.95 ± 0.38	1.36 ± 0.28*	1.31 ± 0.39
Leptin (ng/mL)	5.72 ± 2.78	2.18 ± 1.80*	2.82 ± 0.83*
Adiponectin (μg/mL)	5.90 ± 0.89	5.10 ± 0.98	4.56 ± 0.98*
Corticosterone (ng/mL)	21.94 ± 12.27	257.06 ± 158.2*	26.50 ± 21.43

Data are means ± SD of 9-10 rats per group. *Different from control group, Tukey test, p < 0.05.

### LD experiment

In contrast to the body weight changes in rats consuming CP diets, sleep-deprived animals consuming the LD had only a slow and mild decrease of body weight that reached significance after 48 h when compared to baseline (0 h) [two-way repeated-measures ANOVA group effect, control *vs*. SD: *F*(1,28) = 75.23, p < 0.0001; group × time interaction effect: *F*(4,112) = 34.40, p < 0.0001] (Figure 2C). In contrast, control rats had continuous weight gain (Figure 2C). Repeated ANOVA for body weight changes revealed no differences between the groups [one-way ANOVA group effect: *F*(2,18) = 2.42, p = 0.11] (Figure 2C, left upper panel).

Interestingly, sleep-deprived rats had a sudden increase in food intake starting at 72 h, compared to 0 h and control groups; the control rats had steady food intake [two-way repeated-measures ANOVA group effect, control *vs*. SD: *F*(1,28) = 7.38, p < 0.05; group × time interaction effect: *F*(4,112) = 9.49, p < 0.0001] (Figure 3C). During the rebound period [one-way ANOVA for time effect: *F*(2,24) = 17.31, p < 0.0001], food intake in rebound rats was decreased relative to their consumption at 0 and 96 h (Figure 3C, left upper panel).

There were no differences in the plasma glucose or lactate levels of sleep-deprived rats compared to controls [*F*(2,27) = 0.93, p = 0.40; *F*(2,27) = 0.09, p = 0.90, respectively]; however, unlike the CP diet, the rats fed the LD had comparable stomach weights [*F*(2,27) = 0.62, p = 0.54] among the three groups (Table 5). Glycogen storage [F(2,*2*7) = 24.98, p < 0.0001] was reduced in the SD-96h group, whereas glycogen storage did not differ between the rebound rats and the controls (Table 5). Total cholesterol [F(2,*2*7) = 1.67, p = 0.20] was not modified by SD procedures, but HDL cholesterol [F(2,*2*7) = 12.56, p < 0.001] and triacylglycerol levels [F(2,*2*7) = 10.04, p < 0.001] were reduced in the SD-96h group (Table 5). Although HDL cholesterol levels in the rebound group were maintained at a lower level, triacylglycerol levels in this group were not different than those in the control or SD-96h groups (Table 5).

**Table 5:** Metabolic parameters of control, sleep-deprived for 96 h (SD-96h) and recovery (Rebound) rats fed a liquid diet

PARAMETERS	CONTROL	SD-96H	REBOUND
Stomach weight (g/100 g of body weight)	1.26 ± 0.53	1.27 ± 0.45	1.07 ± 0.39
Liver glycogen (mg/100 mg tissue)	5.56 ± 1.62	2.50 ± 0.99*	5.59 ± 0.39
Glucose (mg/dL)	116.4 ± 12.25	126.4 ± 13.88	121.0 ± 19.62
Lactato (mmol/mL)	2.15 ± 0.70	2.06 ± 0.26	2.14 ± 0.30
Total cholesterol (mg/dL)	76.30 ± 12.42	71.10 ± 8.33	67.60 ± 10.90
HDL cholesterol (mg/dL)	32.85 ± 4.83	25.33 ± 3.61*	25.50 ± 3.61*
Triacylclycerol (mg/dL)	149.9 ± 45.12	65.5 ± 20.06*	109.8 ± 53.70

Data are means ± SD of 9 rats per group. *Different from control group, Tukey test, p < 0.05.

The slight body weight reduction in the sleep-deprived group fed with the LD suggests that their hormonal response to SD-96h was less intense. Consistent with this finding, there were no differences in insulin [*F*(2,27) = 3.32, p = 0.052], ghrelin [*F*(2,27) = 0.25, p = 0.77], or adiponectin levels [*F*(2,27) = 0.32, p = 0.72] between control, SD-96h and rebound rats (Table 6). However, corticosterone and glucagon levels [*F*(2,24) = 32.68 p < 0.0001] were higher and leptin levels [*F*(2,27) = 8.04, p < 0.01] were reduced in SD-96h animals compared to controls (Table 6). After 24 h of recovery, the rebound rats returned to control corticosterone levels, but leptin levels remained decreased. The glucagon levels of rebound rats [*F*(2,27) = 4.61, p < 0.05] were lower than those of SD-96h rats (Table 6).

**Table 6:** Hormonal concentrations of control, sleep-deprived for 96 h (SD-96h) and recovery (Rebound) rats fed a liquid diet

PARAMETERS	CONTROL	SD-96H	REBOUND
Insulin (ng/mL)	1.35 ± 0.76	1.15 ± 0.58	2.16 ± 1.24
Glucagon (ng/mL)	76.97 ± 15.41	105.20 ± 26.82*	72.51 ± 15.75
Ghrelin (μg/mL)	1.07 ± 0.22	0.98 ± 0.18	1.02 ± 0.37
Leptin (ng/mL)	5.72 ± 2.18	3.18 ± 1.43*	2.99 ± 1.12*
Adiponectin (μg/mL)	6.81 ± 1.48	6.36 ± 2.11	6.16 ± 1.03
Corticosterone (ng/mL)	37.64 ± 35.64	265.87 ± 13.47*	34.32 ± 20.14

Data are means ± SD of 9 rats per group. *Different from control group, Tukey test, p < 0.05.

## Discussion

We verified that unbalanced energy metabolism induced by 96 h of SD is related to changes in feeding behavior, which in turn is affected by the type of diet consumed.

Chow-fed sleep-deprived rats presented a starvation-like metabolic and hormonal profile that could not be prevented by the consumption of a high-fat diet with increased energy density. In contrast, such changes were mostly hindered in LD-fed rats.

### Feeding Behavior during 96 h of Sleep Deprivation

Rechtschaffen's group used a hopper specially designed to minimize food spillage to show hyperphagic behavior during long-term SD [7,8]. We have recently developed a procedure to accurately measure food spillage during SD: increases in the amount of food removed from feeders are accompanied by proportional increases in food spillage [19], mainly due to stereotyped gnawing behavior and, to a lesser extent, the environmental conditions imposed by the platform SD technique [25]. After correction for spillage, 96 h of SD in rats fed with CPs induced a transient reduction in food intake and larger body weight decrease. The body weight remained lower even after food intake was reestablished (Figures 2A and 3A).

Likewise, when fed a hypercaloric/high-fat diet, rats had progressive weight loss, suggesting that food intake was decreased (Figures 2B and 3B). Therefore the reduction in body weight is also associated to an increase in energy expenditure promoted by SD. It has been shown that a normocaloric high-fat diet fails to prevent repeated stress-induced hypophagia or weight loss during restraint stress or SD [28]. The decreased food intake of rats fed a high-fat diet was probably masked by the increased gnawing behavior induced by SD, which has been shown to increase the amount of CPs removed from the hopper, especially after 48 h of SD ([19,25] and in this study - data not shown). However due to the texture of high-fat diet, was not possible to recover the crumbs. It is known that high-fat diets produce lower amounts of food spillage than chow pellets, but the non-correction could lead to overestimated values of food intake in these animals, mainly if we considered that the energy content per gram of high-fat diet is approximately 42% higher than chow pellets. Moreover, SD rats fed a balanced LD exhibited only slight weight loss, followed by hyperphagia (Figures 2C and 3C).

In our study, SD using the platform technique induced hyperphagia after 72 h of sleep loss in rats receiving a balanced laboratory LD (Figure 3C). Koban et al. [12] showed that SD increased the expression of the orexigenic neuropeptide Y (NPY) and reduced the anorexigenic peptide proopiomelanocortin (POMC) in the hypothalamus of rats. Such changes have been attributed to the reduction in leptin levels observed after 5 or more days of sleep deprivation [9,10]. Nevertheless, long-term SD of rats on a CP diet induces substantial weight loss, especially through the reduction of adipose tissue [28], which can account for the reduced serum leptin levels. In such cases, increased NPY and the consequent hyperphagia should be a response to weight loss rather than an effect of the SD itself. However, we showed that 4 days of SD leads to increased food intake, NPY hypothalamic expression and weight loss in rats on a LD and that there were no significant changes in the serum levels of insulin, leptin, and ghrelin [13]. We believe that other factors linked directly to SD determine this type of hyperphagia. Although the exact reason for the differences between our results and the previous findings remains unclear, factors such as the feeders used, the diet composition or the strain of rats might account for the discrepancy. To confirm the larger energy deficit in sleep-deprived rats fed with pellets versus liquid food, we verified the metabolic and hormonal responses along with other physiological repercussions of SD.

Interestingly, all groups presented hypophagia during the sleep rebound, probably due to an increased sleep time after the forced sleep loss. Using our protocol, it has been shown that following 96 h of SD the wake time of rats was reduced, whereas the sleep time was increased during the initial 24 h of recovery, especially due to an increase in paradoxical sleep [24]. Since the animals were free to express both feeding and sleep behaviors during the recovery period, it is possible that during this period the sleep mechanisms overcome the hunger mechanisms, consequently reducing food intake.

### Metabolic Deficit in Response to 96 h of Sleep Deprivation

In line with their reduced food intake, SD-96h rats fed with regular CPs or a high-fat diet showed lower stomach weights (Tables 1 and 3). On the other hand, although rats fed with the LD showed an increased food intake after 72 h, their stomach weights at the end of 96 h of SD were no different from those of control animals (Figure 3C, upper panel and Table 5). The absence of increased stomach weight in hyperphagic LD-fed rats is probably related to the faster gastric emptying of liquid versus solid foods; solid and liquid preparations show linear and monoexponential patterns of gastric emptying [29], respectively.

Interestingly, regardless of the diet the rats received or the amount of food they consumed during the last 24 h, we found that liver glycogen stores were reduced after 96 h of SD (Tables 1, 3 and 5). Reduced levels of liver glycogen have been found in different stress models, and they also occur as the result of reduced food intake [30]. Although this finding could justify the lower liver glycogen content in rats fed with CPs and high-fat diets, it cannot explain the reduced liver glycogen in hyperphagic LD-fed rats. Although other factors, such as the stomach emptying or feeding schedules, can affect stomach weight and liver glycogen stores, the SD-induced energy deficit was supported by changes observed in several blood-energy metabolites.

The increased levels of ketone bodies in rats fed with CPs and high-fat diets as well as the decreased triacylglycerol levels in SD-96h rats (regardless of diet consumed) suggest a higher contribution of fat acid oxidation as an energy source. This is a natural body strategy during negative energy balance conditions. Sleep deprivation for 72 to 80 h in humans has also been associated with triacylglycerol decreases and HDL cholesterol increases [31]. Other stress conditions associated with food intake and body weight reduction also result in lowered serum triacylglycerol and ketone body increases [32]. In addition, the loss of triacylglycerol to energy metabolism in the liver could also account for the increased HDL cholesterol observed after 96 h of SD in rats that lose weight; this phenomenon has also been described after fasting-induced weight loss in humans [33].

### Catabolic Hormonal Profile after 96 h of Sleep Deprivation

Compatible with the reduced food intake and large weight decrease in SD-96h rats fed with pellet food (i.e., standard chow or a high-fat diet), we found a number of endocrine changes, including increased levels of orexigenic hormones (such as ghrelin and glucagon) and decreases in the satiety hormone leptin (Tables 2 and 4). Furthermore, in rats fed with CPs, insulin levels were lower in animals sleep deprived for 96 h (Table 2). However, SD-96h rats fed a LD showed only increased glucagon and reduced leptin levels compared with control rats (Table 6).

Previous animal studies have mainly attributed SD-induced hyperphagic behavior to decreased leptin levels [9,10]; however, our present data suggest that other hormones may also play a role. Our experimental design did not allow us determine whether increased glucagon and decreased leptin levels preceded hyperphagic behavior, but it did show decreased liver glycogen stores. More recently, our group demonstrated temporally that increased orexinergic activity during SD activates arcuate NPY neurons, leading to the hyperphagia observed after 72 h of SD. This event was independent of alterations in hormone concentration [13].

Because LD-fed rats showed no changes in glycemia or insulin levels, it is very likely that increased autonomic activity stimulated glucagon release, which led to decreased liver glycogen through increased glycogenolysis. It has been shown that SD using platform [34] and disk-over-water techniques [15] increases the plasma levels of the sympathetic hormones norepinephrine and epinephrine [13], which can also contribute to increased liver glycogenolysis. Therefore, increased sympathetic activity could account for reduced leptin and increased glucagon levels, even in the absence of the substantial body-weight decreases observed in sleep-deprived, LD-fed rats. In the present study, we found that 96 h of SD was highly stressful, and rats under such conditions showed increased corticosteroid levels compared with home-cage controls, regardless of the diet consumed (Tables 2, 4 and 6). Previous studies of paradoxical SD using the platform technique found high corticosterone levels [28,34], which do not always occur in studies using the disk-over-water technique [10,15]. Although the glucocorticoid response can result from the intermediary stress and sleep loss experienced by yoked control animals in disk-over-water studies, we found that corticosterone levels were increased in sleep-deprived rats regardless of the diet consumed. However, pellet-fed and liquid-fed rats were hypophagic and hyperphagic, respectively, suggesting that impaired food intake in pellet-fed rats was due to a factor other than stress itself. This difference is likely due to an easier access of food or a reduction in the gnawing behavior. It is well known that SD increases dopaminergic tonus [34] explaining the increased stereotyped behavior which is inhibited by the absence of chow pellets.

### LD Intake and Body Weight Maintenance under Sleep Deprivation

Perturbations of body energy initiate a coordinated pattern of compensatory intake and expenditure adjustments. Weight decreases from the normally maintained level induce increased energy intake accompanied by decreased daily energy expenditure, thereby blunting further weight loss while providing the conditions for rapid restoration of the lost weight.

Evidence shows that sleep duration plays a role in energy homeostasis by affecting energy expenditure and feeding habits; reciprocally, the fasting-feeding cycle modulates sleep allocation [35].

In humans, sleep loss has been associated with increased risks of obesity and diabetes; however, animal models have failed to reproduce weight gain under SD. Previous studies have suggested that this discrepancy could be due to more extreme SD in experimental rats, a higher resting metabolic rate than humans, and the decreased opportunity to ingest high-calorie foods in the laboratory setting [22]. In the present study, we showed that a highly energy-dense diet failed to prevent weight loss, in agreement with previous descriptions of weight loss in rats receiving high-carbohydrate [36] or high-fat diets [28] during SD. Our finding suggests that the palatability/composition/textures or energy density of the consumed diet cannot account for such disagreement. We also showed that CP-fed rats presented a transient reduction of food intake during the early days of SD that was closely associated with weight loss [19,25].

Some groups have shown that a LD can affect post-ingestive processes, promoting food intake and weight gain in rats and humans [37,38]. Therefore, the facilitative effects of a LD on feeding behavior (e.g., fast gastric emptying [29]) may not only prevent the initial reduction but also allow food intake to increase above the baseline after 72 h of SD to counteract the energy deficit.

## Conclusion

In conclusion, SD using the platform technique induces a wide range of metabolic and hormonal changes that are linked to weight loss in the animal model. Increasing the caloric density of the diet was not sufficient to attenuate these alterations. On the other hand, LD favored food intake, probably by inhibiting the gnawing behavior, consequently lessening the energy deficit induced by SD.

## List of abbreviations

(SD): Sleep deprivation; (SD-96h): sleep deprived for 96 h; (rebound): SD-96h followed by a 24-h recovery; (CPs): chow pellets; (LD): liquid diet; (EDTA): ethylenediaminetetraacetic acid; (RIA): radioimmunoassay; (HDL): high-density lipoprotein cholesterol; (ANOVA): analysis of variance; (CT): control rats; (NPY): neuropeptide Y; (POMC): proopiomelanocortin.

## Competing interests

The authors declare that they have no competing interests.

## Authors' contributions

PJFM, ST and VD'A designed the experimental protocol; PJFM, LF, ACO and VD'A conducted the research and interpreted the data; PJFM analyzed data; PJFM, LF and ACO drafted manuscript; PJFM, LF, ACO, ST and VD'A contributed to revisions of the manuscript; and all authors read and approved the final manuscript and decided to submit the manuscript for publication.
